# Use case driven evaluation of open databases for pediatric cancer research

**DOI:** 10.1186/s13040-018-0190-8

**Published:** 2019-01-15

**Authors:** Fleur Jeanquartier, Claire Jean-Quartier, Andreas Holzinger

**Affiliations:** 1 Institute of Interactive Systems and Data Science, Graz University of Technology, Graz, Austria; 2 Holzinger Group HCI-KDD, Institute for Medical Informatics, Statistics and Documentation, Medical University Graz, Auenbruggerplatz 2/V, Graz, 8036 Austria

**Keywords:** Pediatric oncology, Childhood cancer, Brain tumor, Glioma, Cancer database, Open research, In silico analysis

## Abstract

**Background:**

A plethora of Web resources are available offering information on clinical, pre-clinical, genomic and theoretical aspects of cancer, including not only the comprehensive cancer projects as ICGC and TCGA, but also less-known and more specialized projects on pediatric diseases such as PCGP. However, in case of data on childhood cancer there is very little information openly available. Several web-based resources and tools offer general biomedical data which are not purpose-built, for neither pediatric nor cancer analysis. Additionally, many Web resources on cancer focus on incidence data and statistical social characteristics as well as self-regulating communities.

**Methods:**

We summarize those resources which are open and are considered to support scientific fundamental research, while we address our comparison to 11 identified pediatric cancer-specific resources (5 tools, 6 databases). The evaluation consists of 5 use cases on the example of brain tumor research and covers user-defined search scenarios as well as data mining tasks, also examining interactive visual analysis features.

**Results:**

Web resources differ in terms of information quantity and presentation. Pedican lists an abundance of entries with few selection features. PeCan and PedcBioPortal include visual analysis tools while the latter integrates published and new consortia-based data. UCSC Xena Browser offers an in-depth analysis of genomic data. ICGC data portal provides various features for data analysis and an option to submit own data. Its focus lies on adult Pan-Cancer projects. Pediatric Pan-Cancer datasets are being integrated into PeCan and PedcBioPortal. Comparing information on prominent mutations within glioma discloses well-known, unknown, possible, as well as inapplicable biomarkers. This summary further emphasizes the varying data allocation. Tested tools show advantages and disadvantages, depending on the respective use case scenario, providing inhomogeneous data quantity and information specifics.

**Conclusions:**

Web resources on specific pediatric cancers are less abundant and less-known compared to those offering adult cancer research data. Meanwhile, current efforts of ongoing pediatric data collection and Pan-Cancer projects indicate future opportunities for childhood cancer research, that is greatly needed for both fundamental as well as clinical research.

## Background

The term “Pediatric oncology” represents the branch in medicine concerned with childhood cancer and is defined as “for humans suffering from cancer under the age of 15”. This group can be extended through young adults up to the age of 19.

Pediatric cancer diseases are different from their analogous diseases in adults as shown by studies from the PCGP [1, 2]. The spectrum of mutations which occur in pediatric cancers is different from adult cancers involving a lower mutation rate and frequently single cancer-driving mutations. In comparison, the older group of patients mostly exhibits multiple cancer drivers. Specific mutational characteristics are commonly shared throughout the adult cancer diseases but are different for pediatric cancers. Moreover, the frequency of a particular mutation in pediatric cancer can even vary within the same specific disease depending on age [1, 2].

Knowledge of both, germline as well as somatic mutations in pediatric cancer patients is crucial for the development of novel therapeutics, and personalized medicine based on predisposition [3, 4]. The underlying genomic characterization supports the identification of individual targets across the diversity of cancer diseases as substantial progress in cancer research and clinical cancer care [5]. Cohorts of patients regarding various life-time stages of children different from adults require anti-cancer therapies based on different mechanisms of action [6].

There are numerous different subtypes of cancer and only few children are diagnosed with each type even at high incidence rates, making it difficult to do research on these diseases due to the low number of cases. Nevertheless, future methods including tumor genome sequencing, novel disease models but also expanding resource libraries and reference data will facilitate research on rare cancers [7]. General Web resources on cancer can be found colorredoffering directed and specialized databases [8].

There are well-known large-scale collaborative projects such as the International Cancer Genome Consortium (ICGC) [9], the Cancer Genome Atlas (TCGA) [10] and the Cancer Genome Project (CGP) [11]. Curated data types include information on exomes, whole genome sequences, mutations, mappings and annotations such as the primary site of the tumor at diagnosis. These are foundations for cross cancer analysis. Whereas, data on pediatric cancer is less known and far less available, but essential for research in order to resolve discrepancies compared to adult cancer [1, 12].

Due to collaborative efforts from regional but also global pediatric oncology consortia [13], there are several initiatives for combating pediatric cancer including the Childhood Cancer International with its European Reference Network on Paediatric Cancer [14, 15], the International Society of Paediatric Oncology (SIOP) Europe [16], Australia’s Zero Childhood Cancer research initiative [17], the Physician Data Query (PDQ) Cancer Information from the National Cancer Institute (NCI) [18], the Childrens Oncology Group (COG) [19, 20], Alex’s Lemonade Stand Foundation [21] and others with convening platforms providing information on personalized treatment. Other projects such as the pediatric cancer genome project (PCGP) [1] or the Treehouse Childhood Cancer Initiative which developed its own tumor database [22] focus on the development of novel therapeutics [23] and the fundamental understanding of the disease. Comprehensive cancer projects also implement specialized subgroups focusing on pediatric diseases such as PedBrain within ICGC [24] or the pediatric cancer working group of the American Association for Cancer Research (AACR) and the NCI involving programs such as TARGET [25]. In general, numerous resources incorporating cancer data do exist but there are only a few on pediatric diseases which we have highlighted within the next section.

Still, the quantity of cancer-associated data rises [8] which results in several challenges for sharing information [26]. There is a universal need for interactive data systems targeting clinicians and researchers likewise. Data integration, its visualization and analysis should be accessible but also be provided by the scientific user groups [27, 28]. Morevoer, cancer research has to combine and integrate data of different biological levels towards novel possibilities for computational modeling and simulations [29]. One very important aspect is that many biologists or biomedical researchers are afraid to upload their data into cloud-based databases. Lacking trust among the end-users generally [30] and increasing privacy concerns in the health domain specifically, e.g. due to new European Data Protection Regulations call urgently for new approaches [31]. This is a very difficult and contradictory problem, because on the one hand, cancer researchers deal with sensitive patient data, hence need secure storage, but on the other hand cancer research needs to share and exchange these data without any boundaries. One possible future solution to meet these two contradictory requirements is to make use of federated approaches [32]. Sharing anonymized data may assure privacy. However, most clinical data are of single person trials [33].

### Web resources

**Fig. 1:**
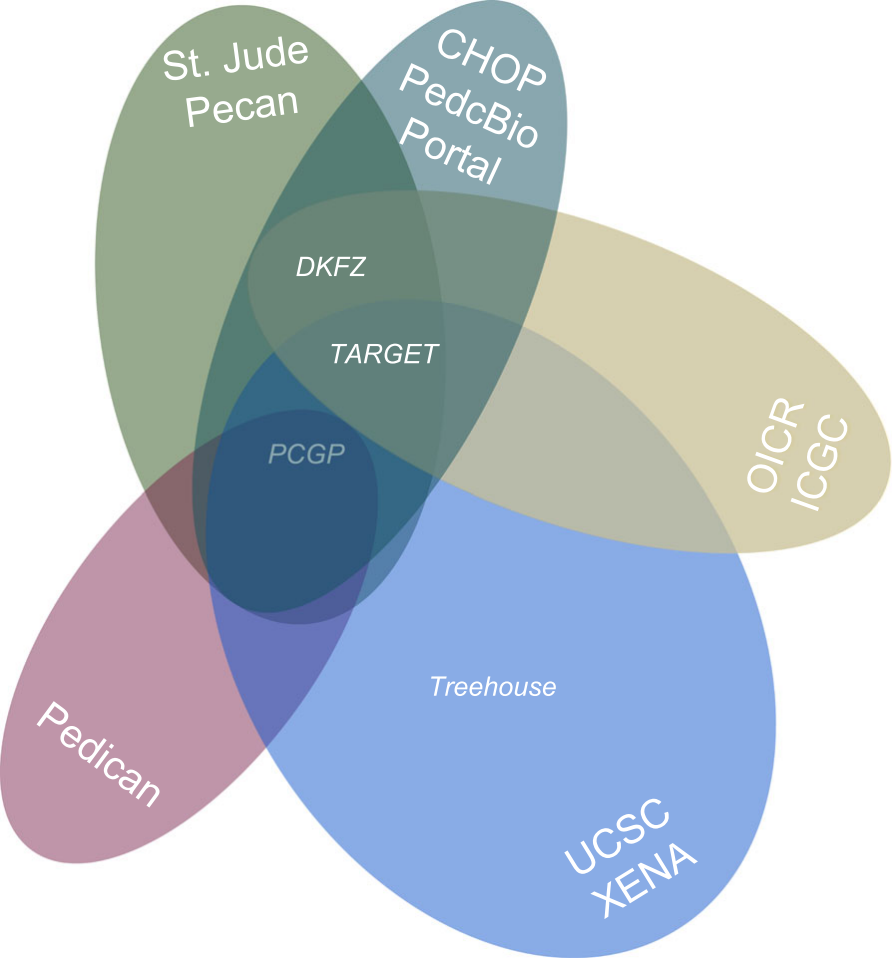
Overview of pediatric cancer databases: Showing tools as venn diagramms with shared datasets

Databases that are focused on pediatric cancers are rare in comparison to the general cancer research community. We identified a few pediatric cancer resources, listed below. Identified resources are further organized into tools and databases. A subset therefrom has been considered to suit the use cases mentioned before, listed in Table 1. The different available databases are integrated into different tools. Tools and the corresponding integration of data are summarized in Table 2. Additionally, Fig. 1 shows, how tools and available datasets are interconnected. The tools and databases are further described below:

**Table 1:** Summary of pediatric cancer web resources, *sorted alphabetically*

Toolname / Url	Maintained by	Databases included	# samples
ICGC Data Portal https://dcc.icgc.org/	OICR	DKFZ/PCBA, TCGA/GDC, TARGET a.o.	4227 donors
PeCan https://pecan.stjude.cloud/	St. Jude	PCGP, DKFZ, TARGET a.o.	4469
PedcBioPortal https://pedcbioportal.org/	CHOP	PCGP, DKFZ, TARGET a.o.	3707
Pedican http://pedican.bioinfo-minzhao.org/	Min Zhao (USC)	PubMed, PCGP, COSMIC a.o.	literature only
Xena Browser https://xenabrowser.net/	UCSC	PCGP, TCGA/GDC, TARGET, Treehouse a.o.	Treehouse PED v8 with 11427 samples a.o.

**Table 2:** Resources and related task completion summary as well as features, *sorted alphabetically by resource name*

	ICGC Data Portal	PeCan	PedcBioPortal	Pedican	Xena Browser
Version info	Data Release 27, 04.30.2018	2015-2018	Version 1.14.1, 2018	2015	03.13.2018
UC 1	Partly (2)	Yes (13)	Partly (3)	Yes (49)	Yes (3)
UC 2	Partly	Partly	Partly	Partly	Partly
UC 3	Yes	Yes	Yes	Yes	Yes
UC 4	Yes	Yes	Yes	No	No
UC 5	Yes	No	Yes	No	Yes
Subtype details	Yes	No	Yes	No	No
Age filter	Yes	No	Yes	No	Possible by phenotype filter
Survival data	Yes	No	Yes	No	Possible by phenotype filter
Enrichment analysis	Gene ontology, pathway targeting compounds	Non-extractable pie charts only for cell cycle, epigenetics, development & signaling	No	Pathway, interactions	Paradigm pathway activity
Alteration type filter	Type, impact, significance	Single-nucleotide variant, insertion/deletions, somatic variant, copy number variation	Type of copy number alteration, listed gene mutation types	Somatic, germline	Somatic mutation assays
Other molecular information	Gene expression, miRNA expression, protein expression, DNA methylation a.o.	Missense, silent, frameshift, exon, nonsense, splice, proteindeletion, intron, untrans-lated region, fusion transcript	Various molecular and phenotypic information	Transcription factor, modifications	Copy number, segments, DNA methylation, RNA sequencing on exon or gene expression
Cancer-related gene filter	Yes (CGC)	No (CGC only)	Mutations noted as to cbioportal cancer genes with 1 or more mutations and all other genes with 2 or more mutations	No	No

#### Tools

- St Jude/Washington University Pediatric Cancer Genomic Data Portal (**PeCan**) aims to provide interactive visualizations of pediatric cancer mutations across various collaborative projects, freely for nonclinical academic research. The data portal makes use of PCGP sequence data, TARGET study data, data from the German cancer consortium (DKTK), its core the German Cancer Research Center (DKFZ) and other institutes as well as samples from St. Jude Children’s Research Hospital itself [34, 35].
- **Pedican**, the online gene resource for pediatric cancers, is a literature-based pediatric gene data resource regarding the pathology of pediatric cancer at the genetic, genomic and epigenetic level [36]. Pedican aims to complement the PCGP project, using mutation information from PCGP, but enriching it with curated data from literature as well as providing annotations regarding information on functions, pathways, regulations and interactions. The tool offers a Web interface for text query, sequence searches, and browsing by highlighted literature evidence.
- The **PedcBioPortal**, a portal for Childhood Cancer Genomics, developed and maintained by a multi-institutional and multinational consortium, is an instance of the genomic data visualization portal cBioPortal. The tool aims to complement genomic pediatric cancer data available such as TARGET with consortia-based research data, providing access to data collected by Children’s Brain Tumor Tissue Consortium, Pediatric Neurooncology Consortium, and St. Baldrick’s Pediatric Cancer Dream Team [37–40].
- The **ICGC** Data Portal offers several data sets as projects including pediatric tumors such as the PedBrain tumor project, coordinated by the DKFZ, which is contributing cancer data with focus on medulloblastoma and subtypes to the International Cancer Genome Consortium (ICGC) [24, 41, 42]. PedBrain Tumor was the first pediatric brain tumor project that contributed to ICGC. Meanwhile, ICGC integrates other projects too, like data from the Children’s Brain Tumor Tissue Consortium (CBTTC). ICGC also integrates TCGA data that is comparable to the Genomic Data Commons (GDC) Data Portal. While GDC is the largest repository of ICGC data, it focuses on studies in the US, whereas ICGC additionally includes data from Canada, EU and others if available. Therefore, this review focusses on the ICGC Data Portal.
- UCSC **Xena** Browser [43] provides genomic data, also some from pediatric cancer samples like Treehouse. Xena hubs allow for integrating both public and private resources.

#### Databases

- **Genomic Resources**
  - The pediatric cancer genome project (**PCGP**) is a collaborative project created by St. Jude Children’s Research Hospital and Washington University School of Medicine. The originally provided data portal “PCGP explore” was based on whole genome sequencing of pediatric tumors with the aim to cover the full spectrum of mutations in pediatric cancers [1]. PCGP is now part of St. Jude PeCan data portal.
  - The Pan-Cancer Study of Childhood Cancers (**PedPanCan**) by the DKFZ includes various sources like ICGC Pedbrain Tumor, PCGP and from Heidelberg and others, and has been integrated into St. Jude PeCan [12].
  - Therapeutically Applicable Research to Generate Effective Treatments (**TARGET**) is a program to provide pediatric cancer data, managed by NCI’s Office of Cancer Genomics. TARGET lists genetic changes that drive the initiation and progression of hard-to-treat childhood cancers [44]. TARGET data is available via the UCSC Xena [43], the GDC Data portal [20] and via the pedcbioportal now on [45].
  - The **Treehouse** Childhood Cancer Initiative is free for any researcher to use, contains RNA-sequencing gene expression data, as well as age, disease and sex [22, 26]. Treehouse Childhood Cancer Projects consolidates datasets under the University of California Santa Cruz (UCSC). The project’s cohort data can be downloaded from UCSC Xena’s Functional Genomics Browser (formerly via the UCSC Cancer Genomics Browser that is no longer under development).
- **Epidemiological Resources**
  - The automated cancer information system (**ACCIS**), developed and provided by the International Agency for Research on Cancer (IARC) of the World Health Organization (WHO), and validated in collaboration with contributing registries. The resource lists data on incidence, occurrence and outcome of various cancers in the young European population [46, 47] and is only available via direct download from the IARC’s Accis homepage as pdf on [48]. By viewing the different tables provided by **ACCIS** for data type “Survival” and “Incidence” we only see a high-level taxonomy of tumor types, categorized after ICCC, where tumors within the CNS are listed under the ICCC Category “III”.
    Some of the incidence and population facts may be used for answering part of the questions only. However, the listed resources could be used for other use cases and, therefore, are included for further inspection.
  - The pediatric oncology group of Ontario network information system (**POGONIS**), childhood cancer database. This resource provides validated data used to monitor incidence and prevalence of childhood cancer, the demand for cancer care, the nature and specifics of cancer treatment, patient outcomes and long-term effects of childhood cancer as well as treatment options [49]. POGONIS does not provide data openly. However, clinicians and researchers can submit a data request to be granted access.

## Methods

In order to compare available databases listed in subsection Web Resources, we take the example of brain tumors, discuss three different problems and thereby address the following questions:

1. Which type of information can be found on (primary) brain tumor subtypes? More precisely, does the web resource include a structured list of specified tumor subtypes? Are theses entries associated to metadata including biochemical or likewise clinical data such as survival or prognosis?
2. Can we extract information on potential glioma biomarkers? Which biomarkers are known, hypothesized, corresponding to classification index for subclasses?
3. Which information can be found on a particular gene by the example of IDH1 coding for isocitratedehydrogenase?
4. What are the most common mutations for childhood glioma? Is further information available on driver mutations?
5. Can the databases be accessed via a web-based application programming interface (API) to suit the task of dynamically accessing and integrating data via web requests into a specific tool?

The use case analysis requires the normalization of relevant terms and concepts for a comparison of the selected resources due to their inhomogeneity of data provision, presentation and inventory. By testing the various case scenarios several possible answers should be taken into account to best compare search results from different databases:

Regarding use case 1 (UC1), we first refer to the Disease Ontology [50] that differentiates “cancer”, “benign neoplasm” and “pre-malignant neoplasm”. Subtypes for brain tumor can be found primarily under “brain cancer”, secondarily under “benign glioma”. Information on the disease named “brain cancer” can be accessed with the *DOID*=1319 for example with EMBL-EBI Ontology Lookup Service [51]. “Brain cancer” has 9 direct subtypes and several synonyms (for example “tumor of the brain”). Regarding benign neoplasm we find “Benign glioma” with 6 child-nodes that can be accessed via *DOID*=0060101 and no related pre-malignant neoplasm.

According to the International Classification of Childhood Cancer (ICCC) [46] there are ependymoma, astrocytoma, medulloblastoma, glioma and specified/unspecified tumor originating from the central nervous system (CNS).

As to UC2 there are different kinds of biomarkers that are disease-related, some that are ideal for disease characterization, as detection and staging, and also some hypothesized examples that could lead to individual therapy. The list of different brain tumor biomarkers ranges from visual, genomic, proteomic and metabolomic [52, 53].

We also question whether there are any statistical visualization output options other than simple bar charts meeting a user’s expectation for more data visualization features [54, 55]. If yes, have there any interactive visual analysis approaches been integrated yet that support the search for possible biomarkers?

In UC3 IDH1 has to be identified as oncogene and to be further associated to several types of malignant brain tumors. Moreover, the user should be informed about relations to possible (drug) targets [56]. A high frequency of mutations in the region of the IDH1 gene has been observed in most of low grade gliomas (LGG) and secondary high grade gliomas (HGG). However, such mutations are less frequently found in pediatric gliomas [57, 58]. Ideally, UC3 yields possible diagnosis strategies as well as a readout on differences between adult and pediatric cancers.

UC4 should highlight possible differences regarding available data. It can also serve as basis for an in-depth analysis of pediatric cancer drivers since one of the primary focuses in cancer research is to identify driver mutations based on computational approaches [59, 60]. Actionable genomic mutations are used for classifcation and targeted therapies [61].

UC5 should highlight possibilities to support data integration insofar as data should be freely and easily accessible via a Rest-API. This application shall examine the suitability for fulfilling the requirement of automatic data retrieval and integration into custom software for supporting data processing and continuing with analysis steps.

## Results

Results are summarized within Table 2 and for UC4 further in Fig. 3 and Table 3. Selected web resources are summarized in regard to their features within Table 2. Several features have been proven useful for exercising the different use case scenarios. Data allocation to disease categories as well as details on comprising disease subtypes is available in some tools only. Additional filtering options regarding the donors age at diagnosis or types of alterations have been integrated to some extent. The availability of processed data is not exclusively limited to cancer gene census (CGC), which lists cancer-implicated mutations by comparison of sequenced data to the reference genome GRCh38.

**Table 3:** Top ten mutated genes within pediatric glioma samples from data amongst several web resources (PedcBioPortal, cBioPortal, ICGC Data Portal, Pecan)

Overall mutation frequency	Gene	Encoded protein	Function
25%	BRAF	B-Raf proto-oncogene	RAS/MAPK signaling for proliferation, differentiation, migration and apoptosis
21%	TP53	tumor protein 53	tumor suppressor, “guardian of the genome”
18%	KIAA1549	UPF0606 protein KIAA1549	transcription regulation
16%	H3F3A	H3 histone family member 3A	DNA accessibility
13%	ATRX	alpha thalassemia/mental retardation syndrome X-linked protein	chromatin remodeler
13%	IDH1	isocitrate dehydrogenase 1	energy metabolism
5%	CDR2	Cerebellar Degeneration Related Protein 2	Myc-regulation
4%	PIK3CA	phosphatidylinositol-4,5-bisphosphate 3-kinase catalytic subunit alpha	phosphorylation, signaling for proliferation and migration
4%	NF1	neurofibromin 1	tumor suppressor
3%	C17ORF47	Chromosome 17 Open Reading Frame 47	uncharacterized protein

The tools’ specific use case suitabilities are further described within the next subsections: In the following subsections results of use cases are summarized individually for each tool.

### Pedican

1. Pedican offers a specific tab to browse cancer types. The search for brain tumor subtypes listed 49 records on entries within Entrez containing further information on genes. Every entry lists sequences and related pathways, literature, gene expression profiles from BioGPS, regulation such as transcription factors or posttranslational modifcations, known variants and various interaction partners. Since this Web resource’s primary focus lies on gene information, there is little information on clinical issues, at most to be found within linked publications.
2. The search for glioma lists 39 entries as described above. Since this list contains all kinds of genes linked to the disease, specific biomarkers cannot be directly extracted from the available information. Certainly, immersion into data on single genes can lead the user to possible biomarkers, however, this process has to be done manually.
3. The query on the gene name lists information for IDH1 as stated above.
4. The query for information on mutations regarding glioma using pedican lists several entries which have to be manually opened in order to extract further mutation information. Browsing for glioma resulted in 39 records. The query option of the mutation search resulted in 47 records. In both cases listed results have to be manually scanned and there is no option for simply spotting most frequent mutations within glioma samples. The category “Pediatric low grade and high grade gliomas” within the tab “browse” lists 4 entries, namely BCL, BCL2L1, BAX and TP53. There is further information on types of mutations, but none on frequency.
  In general, Pedican lists data to genes linking to Web resources such as Entrez and KEGG. It does not offer sophisticated visualization features and only provides tables of global views on gene expression.
5. Pedican offers documentation on how to query PubMed, however, no specific api for programmatic data requests is provided.

### PeCan dataportal

**Fig. 2:**
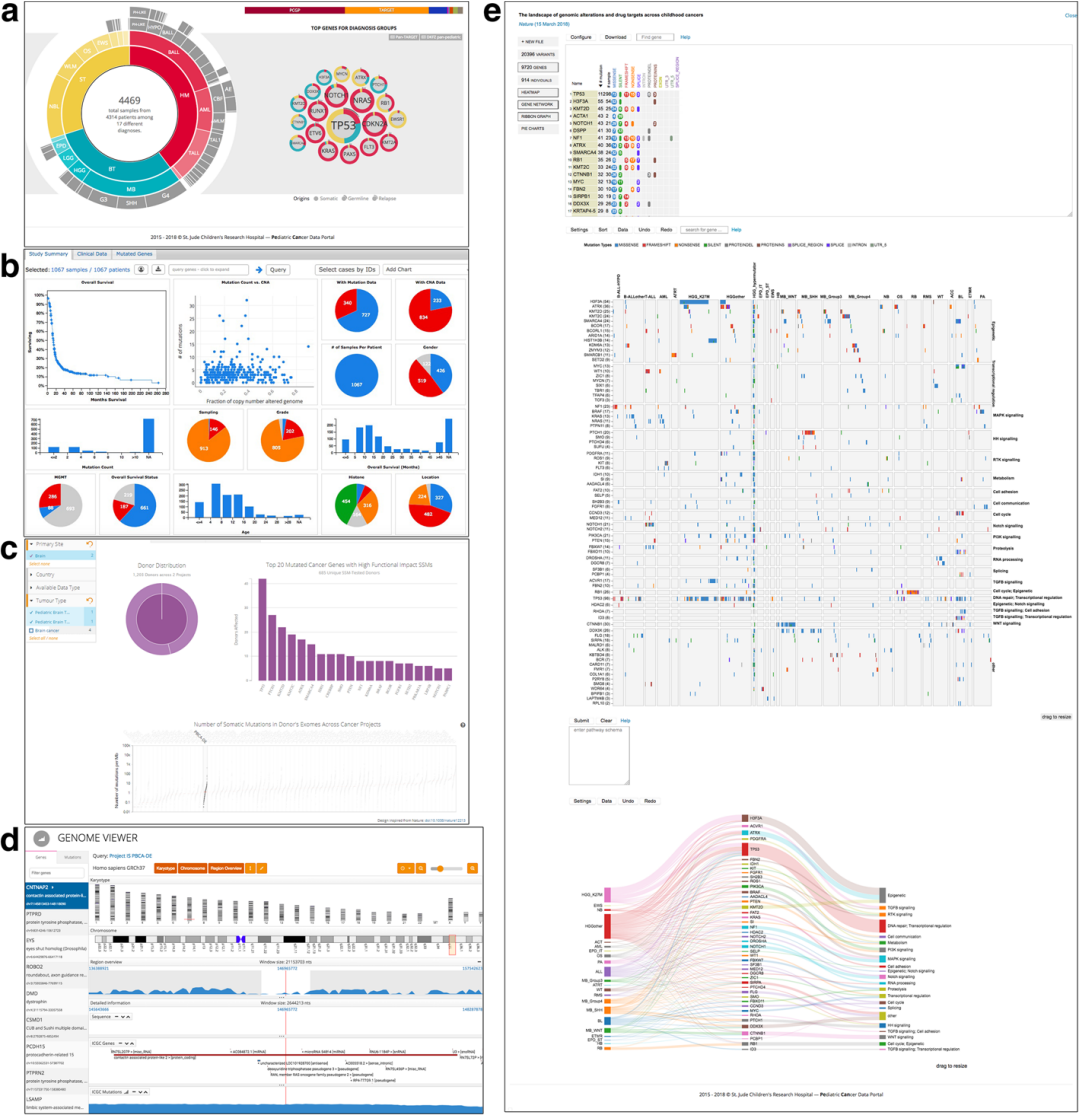
Visualization features: (**a**) PeCan Overview, (**b**) PedcBioPortal Summary View, (**c**) ICGC Summary View, (**d**) ICGC Genome Viewer, (**e**) PeCan Proteinpaint

1. PeCan provides, next to an introductory guide, a glossary including a list of disease types within the help section. ProteinPaint [35], a visual analysis tool for exploring genomic alterations, now found on the left side within the entry site, renders an interactive summary chart of all diseases that displays details to specific disease groups or diseases, compare Fig. 2. By clicking on the visual group of brain tumor or alternatively by searching for a specific disease name, a user can render genetic information specifically for the ones selected.
2. Several study cohorts are found from querying the disease “brain tumor”: While it is difficult to scan through a total of 3045 samples from 2900 patients, a summary of genes with mutations the most found is displayed as bubble chart, sorted by known pathway relations. A user receives more information on the respective gene’s mutations by clicking on a bubble. Within PeCan’s ProteinPaint the sample amount can be further filtered to compare several subtypes as well as specific mutations. Mutation details also include PubMed links that support the task of finding biomarkers.
3. The query on IDH1 reveals 10 mutations for pediatric brain tumor samples (from DKTK, PCGP and BROAD). Mutation details can be viewed by brain tumor subtype, by dataset and detailed information can be found for each containing sample. The mutations found in medulloblastoma, in HGG and in LGG can be analyzed in more detail in terms of pathogenic attributes like class, diagnosis subtype including links to related publications on PubMed, as well as links to NCBI’s single nucleotide polymorphism database.
4. The visual overview of cancer samples, shown in Fig 2, lists glioma with the two subgroups of HGG and LGG within the group of brain tumors. The frequency of mutations can be viewed within the cohort summary as ribbon graph and heat map from selected data. Data on mutated genes from HGG samples are linked to several pathways, which can be selected to search for possible driver mutations, while data from LGG have not been linked yet. HGG results in 3239 mutations with mutated H3F3A and TP53 as the most prominent ones, followed by ACVR1. The case of LGG highlights 195 genes with FGFR1, BRAF and KIAA1549 as the most frequently mutated genes. The tool offers a download button for selected data retrieval.
5. For now, there is only the possibility to upload and download data via data request by a Data Access Agreement during the data request submission. More information available on St. Jude Cloud Documentation are available on [62].

### PedcBioPortal

1. PedcBioPortal distinguishes between adult and pediatric data in the first place. Filtering pediatric data, information on a fraction of studies is listed. When being compared to cBioPortal, PedcBioPortal provides pediatric specific datasets. For example, regarding CNS/brain tumor, both tools provide samples of the PCGP dataset, while PedcBioPortal additionally presents the dataset from “HERBY Clinical Trial, Cancer Cell 2018”, as well as from “ICR London, Cancer Cell 2017”, as the CBTTC and its partners via the Gabriella Miller Kids First Data Resource Center.
2. By selecting a specific tumor type the user can scan through dataset summaries like mutation counts and follow links to PubMed publications for further reading on possible therapeutic developments.
3. IDH1 is found within the pediatric high grade glioma study as one of many mutated genes, and it exhibits only a low mutation frequency. The user also finds a link to additional information on the gene within the protein knowledgebase UniProtKB. By selecting the different samples which contain IDH1 alterations the user finds information on types of mutation. Moreover, the user can compare alteration occurrences of different genes among samples by using a clusterable heatmap visualization. Mutation details include information on the protein encoded by IDH1, for example its 3D structure and a link to the RCSB protein data bank. Moreover, an overall survival Kaplan-Meier estimate can be printed, that shows survival rates of cases with and without alterations in the queried gene. Further network visualization and analysis of this gene provides information on possible drug targets.
4. In order to receive an overview of mutations for specific cancer diseases, individual studies have to be selected. Nine studies are available on pediatric glioma, while 18 studies on adult and pediatric data can be accessed. Study details are listed as interactive column allowing data to be sorted for instance by number of mutations. These data have to be further filtered by age, via the respective column, in order to receive data from pediatric samples only. Results on gene mutation frequency highlight TP53, H3F3A and ATRX. CBioPortal for Cancer Genomics provides visualization and analysis for PedcBioPortal. Still, if the public site of cBioPortal is directly used instead of PedcBioPortal, the query for pediatric glioma results in different top mutated genes, in fact, BRAF, KIAA1549 and IDH1. CBioPortal allocates data from only a subset of the data integrated in PedcBioPortal.
5. PedcBioPortal offers a web api to perform queries from the different pediatric studies instanced from cBioPortal [63]. With requesting http://www.cbioportal.org/webservice.do?cmd=getTypesOfCancerone may loop through a list of cancer types such as listing only those that are glioma related, while querying http://www.cbioportal.org/webservice.do?cmd=getMutationData&case_set_id=gbm_tcga_all&genetic_profile_id=gbm_tcga_mutations&gene_list=IDH1+TP53will request a set of mutation data with several details such as type, status, chromosome, start and end position among others.

### ICGC data portal

1. Various pediatric tumor data, such as “PedBrain Tumor” can be viewed in **ICGC’s Data Portal** [64]. Thereby the project summary reveals that its data focuses on pediatric brain tumors, in particular on the two types of medulloblastoma and pediatric pilocytic astrocytoma. No information on a comprehensive set of subtypes is given.
2. The project’s overview page shows the top 20 mutated cancer genes. The summary holds PubMed links to related information on epigenetic subgroups and subgroup biomarkers, driver mutations, as well as on biomarkers for specific pathway activations. These are good starting points to find suitable biomarkers.
  Moreover, the data portal’s advanced search offers multiple options on filtering the project data, ranging from donors, genes up to specific mutation filters. Having a closer look at the meta-data on donors, next to mutation counts, the donors’ age, stage and survival days are further indicators to deepen the search for biomarkers. Each listed mutation can further be checked for a detail view, providing information on consequences, cancer distribution, protein and genomic context. Additional links include the integrated genome viewer as well as external links to further information on Ensembl.
3. IDH1 appears within the top 20 mutated cancer genes in the project summary view of the PedBrain Project. The project’s related publications include information on IDH1 mutations to be rare for childhood glioblastoma. By clicking on the gene symbol further information such as reactome pathways, gene ontology terms, protein information but also the cancer distribution is displayed. Cancer distribution shows that mutated IDH1 is mainly found in brain cancer, in particular within LGG and only to a low extent in medulloblastoma. Filtering of gene IDH1 results in only 13 mutations in 20 donors out of 554 donors. The number of samples can be computed via downloading the raw data and filtered by the selected donor IDs.
4. An option to filter age-related data has been included into this resource under the donor section. This allows the user to isolate data on pediatric glioma within the ICGC data portal. Selecting all available entries for pediatric brain tumors highlights IDH1 as the most prominent example of genes affected by mutations within the young cancer patients, followed by CDR2 and ATRX. Mutation counts can be normalized to the number of donors, while the number of individual samples can be only extracted manually from downloadable raw content. These results are visualized as interactive bar chart rendering further information on individual samples as well as the gene by hovering over or clicking on selected bars.
5. Data which is available via ICGC’s data portal can be accessed via ICGC’s api that provides curl as well as https get requests. Information on the api can be found at [65] as well as via python rest services [66]. For example by accessing https://dcc.icgc.org/api/v1/projects/PBCA-DE/mutationsthe user receives a list of mutation information within the specified pediatric brain tumor project. Several filter parameters can be added to specify a particular query. ICGC’s web user interface allows for complex queries, due to it’s Portal Query Language (PQL) [67]. Requesting https://dcc.icgc.org/search/g?filters={"donor":{"projectId":{"is":["LGG-US","GBM-US","GBM-CN"]},"primarySite":{"is":["Brain"]},"ageAtDiagnosisGroup":{"is":["10-19"]}}}&donors={"from":1}&genes={"size":10,"sort":"donorsAffectedFiltered","from":1} lists top ten mutated genes for pediatric brain tumors.

### UCSC Xena browser

1. The query on glioma within datasets from the Treehouse study and others can be executed manually after downloading the expression data or by using the visualization tools provided by **UCSC**. When selecting the Treehouse dataset, last updated July 2018, as search object within the **Xena browser**, a list of the disease phenotypes reveals an incomplete list of tumor types which contains 3 glioma subtypes. Now a user may ask how to define brain tumor subtypes from a list of several cancer disease types. Therefore, we also searched within the raw data provided as tab separated values which can be sorted by the phenotypic data type “disease” that revealed a result of 6 brain tumor related types.
2. Searching for biomarkers can be done via sorting gene expression count on certain disease types.
3. Information on IDH1 can be found with the help of UCSC toolset. For example, searching for IDH1 in UCSC visgene results in a brain image of a mouse. The search within the Treehouse public expression dataset reveals a higher occurrence of IDH1 within the diffuse intrinsic pontine glioma (compared to glioma, glioblastoma multiforme and gliomatosis cerebri). Unfortunately, the tumor map [43] does not find IDH1, whereas the Xena browser’s heatmap view yields frequency and variance differences for IDH1 depending on the age. Moreover, the analysis of another Treehouse dataset on neuroblastoma showed that copy number variations are higher in undifferentiated or poorly differentiated cells.
4. UCSC Xena’s Heatmap Visualization tool allows for manual selection of specific genes only. Therefore, it is easier to sort the downloadable spreadsheet data file. However, there is no column on mutations within the dataset. The genome browser with focus on annotation tracks does neither provide any mutation ranking.
5. Treehouse data can be accessed via UCSC tools in three ways: First, via direct download, second via UCSC Xena on [68] and third via the newer Xena interfaces with python [69].

## Discussion

Every Web resource has its own strengths and target users. It has to be kept in mind that few use cases only cannot fully grasp the potential of each single resource available. However, the above described use cases indicate the utility of the tools discussed.

In the first use case the result of 49 entries that list different types and subtypes of brain tumors clearly distinguishes Pedican from the other tools. Still, this number does not relate to data quantity and information provided by the individual items. In the second use case, our search for possible biomarkers could be best supported by integrated visual analysis tools, such as Pecan’s ProteinPaint and PedcBioPortal, as well as linking to literature, such as provided by Pedican, PeCan and PedcBioPortal.

The third use case could be successfully performed by using the different tools’ feature-sets. PeCan as well as PedcBioPortal foreground a gene search with visual summaries and multiple related information.

**Fig. 3:**
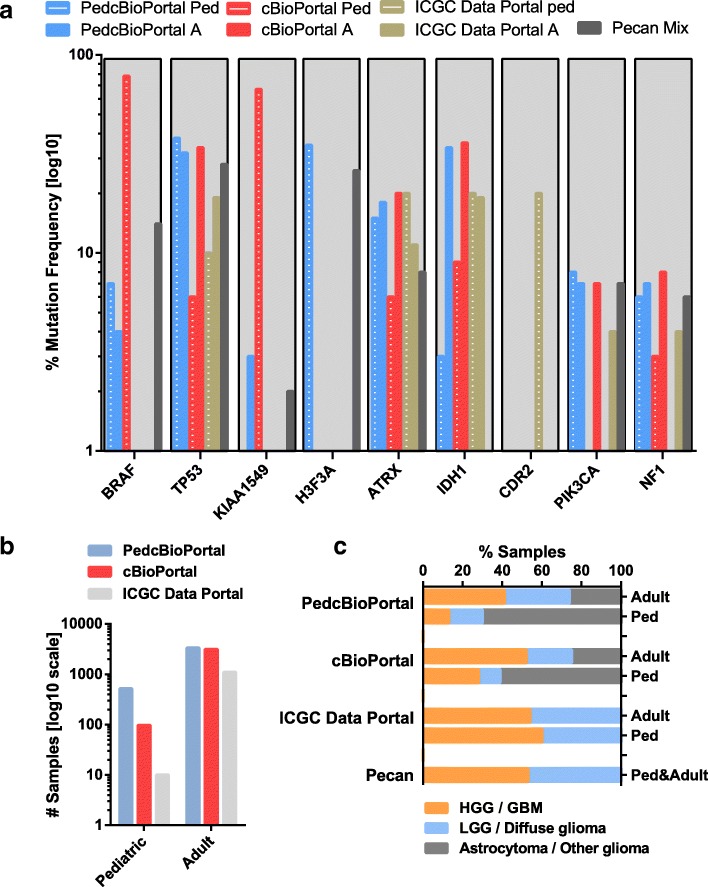
Data allocation on glioma samples by different tools. **a** Frequency of mutated genes for pediatric and adult glioma: Comparison of the main web tools which allow for distinct age-related query. Selected gene mutation count over sample count within data sets on pediatric (Ped, age 1-19) and adult (A, age 20+) cancers or mixed samples without age-distinction (Mix) provided by PedcBioPortal (blue), cBioPortal (red), ICGC Data Portal (brown, mutation count normalized over number of donors instead of samples) and Pecan (grey). **b** Number of samples on glioma: Data provision for separate selection of pediatric and adult glioma samples by the three web resources PedcBioPortal (blue), cBioPortal (red) and ICGC Data Portal (grey), results listed in log(10) scale. **c** Differences in data quantity on glioma subtypes: Percentage of samples on high-grade glioma (HGG) or glioblastoma (orange), and low-grade glioma (LGG) or diffuse glioma (light-blue) and further non-specified glioma and related astrocytoma (grey), with initial diagnosis at the age below 20 (Ped) or 20+ (Adult) provided by PedcBioPortal, cBioPortal, ICGC Data Portal and Pecan

Figure 3 shows a summary of UC4 and highlights the inhomogeneity of available data-sets within the web resources. In order to observe variations between pediatric and adult cancer, the overview of results for UC4 are further supplemented by likewise available data on adult cancers. Those resources offering mainly pediatric data-sets do hardly include higher numbers on mutations within IDH1 while in adult samples the mutated gene is known to be connected to glioma [58]. This aspect is best shown by the direct comparison of PedcBioPortal and cBioPortal. Further differences of gene mutation counts result from the availability of diverging data within the various resources, as shown in Fig. 3b. Figure 3c particularly demonstrates data provision related to cancer subtypes. PedcBioPortal offers a balanced subset of data between pediatric and adult HGG & LGG and undefined glioma. Whereas, cBioPortal provides mainly data on adult HGG & LGG compared to pediatric astrocytoma. Pecan offered to query HGG or LGG while there was no option to query general glioma.

Table 3 lists the average top ten mutated genes within pediatric glioma from the selected databases of PedcBioPortal & cBioPortal, ICGC Data Portal and Pecan. These selected resources supported the process of querying pediatric donors. Still, pecan lacks specific filtering options. The list of genes as visualized in in Fig. 3a, again reflects the inhomogeneity of data provision by the various databases, since the selected resources differ greatly in data quantity on cancer subtypes which are associated to discriminative gene-mutations and further biomarkers. These discrepancies mainly originate from two aspects, first data resources, and secondly data allocation. First aspect could appeal for more publicity of data repositories and clinical data upload as standard practice. Second aspect calls for standard structuring of data and query options as e.g. cancer subtype classification according to ICD.

UC5 outlines the differences regarding the availability and extent of web-based APIs between current tools. Most of them do not offer any REST-ful service or similar possibilities for an easy integration of provided data. Only PedcBioPortal with cBioPortal offers a webservice interface providing many different methods that can be used for getting data such as a list of cancer types, information on genes or clinical data. Unfortunately, St. Jude’s PeCan does not provide any API, yet. However, since PedcBioPortal integrates data from various projects and studies, data from PCGP, DKFZ as well as TARGET are also available via PedcBioPortal’s API.

Ease of use is another matter of concern which renders a resource more or less suitable for respective groups of users. So far, mainly literature curated information is used by clinicians manually. Supporting simple integration and reuse of such data is specifically important for this user group as it also requires a higher level of usability. Pedican offers a comprehensive tutorial with step-by step guides and various search options for entries such as gene names, literature, mutations an other annotations. Unfortunately, the project team is small and and manual curation needs time, therefore database updates are annually [36] at most. St. Jude’s and Washington University’s cooperation on PCGP depicts a project as an international effort for a deeper understanding of cancer driving mutations and underlying alterations of signaling pathways. Researchers and clinicians are able to explore data from PCGP with St. Jude’s Cloud or PeCan Data Portal, but also with other tools as listed in Fig. 1. St. Jude’s Pecan Data Portal offers several visualization features regarding pediatric cancer mutations as tutorials for its tools Protein Paint and Pecan PIE. PedcBioPortal provides a feature-rich user interface for childhood cancer research. We believe that its integrative approach between already published and new consortia-based data is an important step towards making more data available on pediatric cancer. However, we indicate certain shortcomings. Regarding a gene’s mutations analysis, someone has to have an idea which gene to choose for comparison and there are no obvious selection suggestions provided. Other shortcomings relate to performance and integration issues. The feature set of cBioportal with its visual analysis tools is steadily being enhanced owing to its growing community, but a user has to wait many seconds for several visualization renderings. Moreover, a pediatric cancer researcher is limited to data on the small subset of patient samples available. These facts underline the necessity of fostering integrativeness and visual support to analysis tools [70]. The ICGC data portal offers numerous possibilities for data analysis, including an overview visualization, see also Fig. 2, as well as a detailed visualization and phenotype comparison. There is the option to submit data to the Europen Genome-Phenome Archive (EGA) [71]. File specifications for clinical data submission include templates for optional donor biomarker files. Template structure includes annotation options describing whether a biomarker test was positive or not and an optional threshold value indicating positive results. However, no such data has been part of the PedBran Tumor project, yet. ICGC’s Pan-Cancer Analysis of Whole Genomes (PCAWG) focuses on adult cancers in general. No in size comparable pediatric pendant has been published, yet. However, the DKFZ is already working on Pediatric Pan-Cancer datasets [12, 72] which are being integrated into PeCan, PedCBioPortal and have been visualized within DKFZ’s R2 Genomics Platform [73]. Pan-cancer analysis enables the identif tication of distinct features between childhood and adult cancer [12].

There are ongoing efforts to integrate data from childhood cancer studies into existing tools, such as for the example of UCSC providing Treehouse study data. Furthermore, the Center for Data-Driven Discovery in Biomedicine at Children’s Hospital of Philadelphia (CHOP) is not only the new provider for PedcBioPortal but also leading a new pediatric data resource center for research in childhood cancer appealing to join forces [74]. Again, the Kids First Data Resource Center will be publicly released as beta version within late 2018, providing a cloud-based data-sharing infrastructure combined with data visualization tools, intended to be used by the related world-wide research community. It is hosted by CHOP, amongst others, it is also led by the NCI and includes data from CBTTC, the Pacific Pediatric Neuro-Oncology Consortia (PNOC) and affiliates [75].

Generally, such pediatric cancer tools that integrate multiple study data are of great importance, as comprehensive genetic diagnostics in children support effective targeted therapies [12, 76]. Cooperative efforts are needed to get comparable results [77]. Much more data is needed to be openly available in order to support and stimulate research, e.g. by fostering the integration of biobanks [78] and to make data, algorithms and tools open to the international research community, e.g. through the European Open Science Cloud [79]. Other projects also call for an establishment of a freely accessible aggregated biomedical database of pediatric data [80]. Storing whole genome sequences from children on a regular basis by conducting newborn sequencing with parental consent [81] could lead towards a better understanding and early as well as rapid detection of pediatric diseases.

## Conclusions

Every tool listed above offers specific features of its own having certain advantages and disadvantages. The most suitable resource depends on the respective question in mind. A high level of data provision and its adequate allocation is fundamentally important for finding answers to a particular question in mind. In order to increase the availability of data, researchers have to be familiar with these web resources and therefore be made aware to share and upload their new data on pediatric cancers. So far, resources which hold data on specific pediatric cancers are rare compared to those offering adult cancer research data. Our evaluation results could prove useful for answering questions as to where are we now, where can we find published data and where can we contribute with new data. Current efforts indicate future opportunities for childhood cancer research to get more awareness, focus and impact within cancer research. Future investigations into specific differences of pediatric and the corresponding adult cancer may lead to novel therapy approaches. There is a need for cooperative efforts providing big data in Pediatrics to support decision making. Generally, cancer research needs international cross-domain cooperation in a joint effort without boundaries.

## Abbreviations

AACR: American association for cancer research
ACCIS: Automated cancer information system
API: Application programming interface
ATRX: Alpha thalassemia X-linked protein
CBTTC: Children’s brain tumor tissue consortium
CGC: Cancer gene census
CGP: Cancer genome project
CHOP: Children’s hospital of philadelphia
CNS: Central nervous system
COG: Childrens oncology group
DKFZ: Deutsches Krebsforschungszentrum
DKTK: German cancer consortium
DO: Disease ontology
EGA: Europen genome-phenome archive
FGFR: Fibroblast growth factor receptor
GDC: Genomic data commons
H3F3A: H3 histone family member 3A
HGG: High grade glioma
IARC: International agency for research on cancer
ICCC: International classification of childhood cancer
ICGC: International cancer genome consortium
IDH: Isocitrate dehydrogenase
LGG: Low grade glioma
NCI: National cancer institute
NF: Neurofibromin
PCAWG: Pan-cancer analysis of whole genomes
PCGP: Pediatric cancer genome project
PDGFRA: Platelet derived growth factor receptor alpha
PDQ: Physician data query
PeCan: Pediatric cancer genomic data portal
Pedican: Pediatric cancer gene database
PIK3CA: Phosphatidylinositol-bisphosphate-3-kinase catalytic subunit alpha
PNOC: Pacific pediatric neuro-oncology consortia
POGONIS: Pediatric oncology group of ontario network information system
PTEN: Phosphatase and tensin homolog
REST: Representational state transfer
TARGET: Therapeutically applicable research to generate effective treatments
TCGA: The cancer genome project
TP53: Tumor protein 53
UC: Use case
UCSC: University of California, Santa Cruz
USC: University of the sunshine coast
WHO: World health organization
